# Gene Expression and Molecular Characterization of a Xylanase from Chicken Cecum Metagenome

**DOI:** 10.1155/2017/4018398

**Published:** 2017-07-02

**Authors:** Hind AL-Darkazali, Vithaya Meevootisom, Duangnate Isarangkul, Suthep Wiyakrutta

**Affiliations:** ^1^Department of Microbiology, Faculty of Science, Mahidol University, Ratchathewi, Bangkok 10400, Thailand; ^2^Chemical and Petrochemical Research Center, Commission for Research and Industrial Development, Ministry of Industry and Minerals, 10068 Baghdad, Iraq

## Abstract

A xylanase gene* xynA*_*MG1*_ with a 1,116-bp open reading frame, encoding an endo-*β*-1,4-xylanase, was cloned from a chicken cecum metagenome. The translated XynA_MG1_ protein consisted of 372 amino acids including a putative signal peptide of 23 amino acids. The calculated molecular mass of the mature XynA_MG1_ was 40,013 Da, with a theoretical pI value of 5.76. The amino acid sequence of XynA_MG1_ showed 59% identity to endo-*β*-1,4-xylanase from* Prevotella bryantii* and* Prevotella ruminicola* and 58% identity to that from* Prevotella copri*. XynA_MG1_ has two conserved motifs, DVVNE and TEXD, containing two active site glutamates and an invariant asparagine, characteristic of GH10 family xylanase. The* xynA*_*MG1*_ gene without signal peptide sequence was cloned and fused with thioredoxin protein (Trx.Tag) in pET-32a plasmid and overexpressed in* Escherichia coli* Tuner™(DE3)pLysS. The purified mature XynA_MG1_ was highly salt-tolerant and stable and displayed higher than 96% of its catalytic activity in the reaction containing 1 to 4 M NaCl. It was only slightly affected by common organic solvents added in aqueous solution to up to 5 M. This chicken cecum metagenome-derived xylanase has potential applications in animal feed additives and industrial enzymatic processes requiring exposure to high concentrations of salt and organic solvents.

## 1. Introduction

Microbial enzymes have been recognized as a major source of various types of biocatalysts which can be successfully applied in different industrial processes [1]. To be practically useful and economically competitive, industrial enzymes must display high activity and stability under harsh conditions to help reduce the production costs [2]. Carbohydrases, enzymes that degrade polymeric carbohydrates, are currently employed in various industries including food and beverages, detergent, biofuel production, textile, paper and pulp, leather industries, and animal feed. However, there are only two enzymes, xylanase and glucanase that dominate the global enzyme market by more than 80% [1, 3].

Xylanases (endo-1,4-beta-xylanase; EC 3.2.1.8) are glycosidases which randomly cleave internal*β*-1,4-D-xylosidic linkages of xylan [4], a bioheteropolymer consisting of D-xylose homopolymer backbone that can be substituted to diverse degrees with glucuronosyl, 4-*O*-methyl-D-glucuronopyranosyl, *α*-L-arabinofuranosyl, acetyl, feruloyl, and/or* p*-coumaroyl residues and is one of the most abundant polysaccharides in nature [5–7]. Complete depolymerization of xylan needs a synergistic action of several xylanolytic enzymes. Majority of the xylanases are confined either to glycosyl hydrolase family 10 (GH10) or to family 11 (GH11) based on similarities in their hydrophobic clusters and amino acid sequences of the catalytic domains. However, some are found in other glycosyl hydrolase families including 5, 7, 8, 16, 26, 43, 52, and 62 [4, 8, 9].

Generally, intestinal microorganisms of plant-eating animals are known to be excellent sources of various hydrolytic enzymes. Considering that poultry feeds consist mainly of cereal grains which are rich in nonstarch polysaccharides including xylans and arabinoxylans, microorganisms producing nonstarch polysaccharide hydrolases including xylanase should be abundant in chicken intestine. Chicken cecal microbiota has been shown, in a culture-based study, predominated by the phyla* Bacteroidetes* and* Firmicutes* [10]. However, majority of gut microorganisms are unculturable. Therefore, metagenomic strategy is used to recover the genes of desired enzymes [11]. Recently, a metagenomic study of chicken cecal microbiome showed that up to 1.5% of the sequences represented glycosyl hydrolase (GH) domains with over two hundred different sequences of nonstarch polysaccharide-degrading enzymes found [12].

In this paper, we report the gene cloning, sequence and phylogenetic analyses, structural prediction, heterologous expression, and molecular and catalytic characterizations of a new GH10 family endo-xylanase derived from a chicken cecum metagenome.

## 2. Materials and Methods

### 2.1. Strains, Plasmids, and Chemicals

The* Escherichia coli* EPI300™-T1^R^ clone harboring fosmid pCC1FOS carrying a chicken cecal metagenomic DNA fragment containing a xylanase gene was a gift from Dr Kenneth van Driel. All enzymes and dNTPs in this study were purchased from New England BioLabs Inc., USA, and Promega, USA. Plasmid DNA extraction and purification kit was purchased from GE Healthcare, UK. TALON Superflow Metal Affinity Resin (Clonetech) was purchased from TaKaRa (Otsu, Japan). The expression vector pET-32a (Novagen) was used for cloning and expressing the xylanase.* E. coli *Tuner (DE3)pLysS was used as expression host and was cultivated on Luria–Bertani medium (Difco). The enzyme substrates used were xylan from oat-spelt (Fluka), xylan from beechwood (Megazyme), *α*-cellulose (Sigma), carboxymethyl cellulose (Sigma), starch (Sigma), *β*-glucan from barley (Sigma), 4-nitrophenyl-*β*-D-xylopyranoside (Megazyme), 4-nitrophenyl-*β*-D-cellobioside (Sigma), and 4-nitrophenyl-*α*-D-galactopyranoside (Fluka). Molecular weight standard mix containing xylose, xylobiose, xylotriose, xylotetraose, xylopentaose, and xylohexaose (Megazyme) was the gift from Professor Khanok Ratanakhanokchai, KMUTT, Thailand. All other chemicals were obtained from Sigma-Aldrich (St. Louis, MO, USA).

### 2.2. Bioinformatic Analysis of DNA and Amino Acid Sequences

Nucleotide sequence recognized as xylanase gene was translated into amino acids. Similar sequences were retrieved from the GenBank database using the BLAST search. Sequence alignment and phylogenetic analysis were done using the CLC Main Workbench 7.7 sequence analysis software package (CLC bio). To determine the family of the xylanase, the position of glutamate residues of the active site, and the highly conserved motifs of GH10, the ExPASy–PROSITE (http:/www.expasy.org/prosite) was used. The signal peptide was predicted by using SignalP 4.1 server (http://www.cbs.dtu.dk/services/SignalP). The theoretical pI and molecular weight were predicted using an online prediction tool (http://www.expasy.org/tools/pi_tool.html). The structure of XynA_MG1_ protein was predicted using the SWISSMODEL (https://swissmodel.expasy.org/) and IntFOLD (http://www.reading.ac.uk/bioinf/IntFOLD/) servers. Images were generated using PyMOL software (http://www.pymol.org/).

### 2.3. Construction of pET32a-XynA_MG1_ Plasmid, Gene Expression, and Purification of XynA_MG1_ Xylanase

The xylanase gene* xynA*_*MG1*_ was amplified by PCR from the pCC1FOS fosmid clones, using the Xyl524GH10CH-Fw: 5′-ATGAGCTCGCTGACACCC-3′ forward and Xyl524GH10CH-Rv: 5′-CTAAGCTTGTCACTGCTTGAAC-3′ reverse primers. The primer pair targeted the truncated gene, excluding the leader peptide encoding sequences, and introduced* Sac*I and* Hind*III restriction sites at 5′- and 3′-end of the gene. The amplified PCR product was digested with* Sac*I and* Hind*III and ligated, using T4 DNA polymerase, into pET-32a(+) vector previously linearized with the same restriction enzymes and dephosphorylated with Antarctic Phosphatase (New England BioLabs). The expression plasmid (pET32a–*xynA*_*MG1*_), having the* xynA*_*MG1*_ gene under the control of the T7 promoter, was used to transform chemically competent [13]* E. coli* Tuner (DH3)pLysS cells to give* E. coli* Tuner (pET32a–*xynA*_*MG1*_) expression strain. Positive clones were proven by colony PCR and the presence of xylanase activity in the cell lysate. Selected* E. coli* Tuner (pET32a–*xynA*_*MG1*_) clone was grown in LB broth containing 34 *μ*g/mL and 100 *μ*g/mL of chloramphenicol and ampicillin, respectively, incubated at 37°C with 200 rpm shaking until the culture reached an OD_600_ nm of 0.6. Then the* xynA*_*MG1*_ gene expression was induced by adding IPTG to a final concentration of 0.4 mM and the culture was further incubated for 5 h at 37°C with 200 rpm shaking. The cells were harvested by centrifugation, resuspended in nine volumes of ice-cold phosphate buffer (50 mM sodium phosphate, 1.2 M NaCl, 10 mM imidazole) pH 7.2, and subjected to ultrasonic cell disruption while keeping in an ice-bath. Unbroken cells and insoluble cell debris were removed by centrifugation at 12,000*g* for 25 min yielding the clear soluble cell lysate. The cell lysates were subjected to sodium dodecyl sulphate polyacrylamide gel electrophoresis (SDS-PAGE, 12% separating gel) to determine the expression profile of the XynA_MG1_ xylanase. The recombinant XynA_MG1_ was purified from cell lysate by Immobilized Metal Affinity Chromatography (IMAC) using the TALON Metal Affinity Resin (Clontech Lab, Inc.) charged with cobalt. The IMAC column (Tricon 10 × 100 mm) was operated using an ÄKTA purifier FPLC system (GE Healthcare Bio-Sciences) at the 0.2 mL/min flow rate. The bound proteins were eluted with 10 bed volumes of linear gradient of 1–150 mM imidazole in phosphate buffer (50 mM sodium phosphate, 1.2 M NaCl) pH 7.2, at 0.5 mL/min flow rate. Fractions containing xylanase activity were pooled and concentrated by the Amicon® 30 kDa cut off centrifugal filter device (EMD Millipore) at 4,000 rpm, 4°C. The concentrated XynA_MG1_ was repurified using the IMAC and then desalted as described above. The pET-32a derived Trx-His*∙*tag which fused to the N-terminus of the expressed XynA_MG1_ was removed by digestion with enterokinase (New England BioLabs) for 16 h at 23°C. The digestion mixture was then loaded onto the IMAC column as above, and the recombinant XynA_MG1_ was retrieved from the flow-through, while the Trx-His*∙*tag and the uncleaved Trx-His-XynA_MG1_ remained bound to the TALON resin. Protein concentration was determined by the Bradford method using bovine serum albumin (BSA) as the standard. The purity of XynA_MG1_ was analyzed on SDS-PAGE. The zymogram analysis was done on 12% gel native PAGE containing 0.2% oat-spelt xylan as the substrate [14].

### 2.4. Biochemical Characterization of Purified XynA_MG1_ Xylanase

Substrate specificity of XynA_MG1_ was determined by assaying activity towards different substrates at 1% (w/v) concentration in 50 mM citrate buffer pH 5.5. The tested substrates were of the following polymers: beechwood xylan, oat-spelt xylan, *α*-cellulose, carboxymethyl cellulose, starch, and barley *β*-glucan [14, 15]. The xylanase activity was estimated by measuring reducing sugar released from the reaction using the DNS method [16]. XynA_MG1_ was also tested against 4 mg/mL of 4-nitrophenyl-*β*-D-xylopyranoside, 4-nitrophenyl-*β*-D-cellobioside, and 4-nitrophenyl-*α*-D-galactopyranoside synthetic chromogenic substrates as previously described [17]. End products from XynA_MG1_ catalyzed hydrolysis of beechwood xylan and 4-nitrophenyl-*β*-D-xylopyranoside were analyzed by thin-layer chromatography (TLC) on a Silica gel 60 F_254_ plate (10 × 10 cm) (Merck, Darmstadt, Germany) using a developing solvent (chloroform/acetic acid/water 6 : 7 : 1, v/v). The products were visualized by spraying with ethanol/sulfuric acid (95 : 5, v/v) and heating for 10 min at 100°C as previously described [18].

### 2.5. Effect of pH and Temperature on XynA_MG1_ Activity and Stability

To determine the optimum pH for XynA_MG1_ activity, the enzyme was allowed to function in buffers ranging from pH 3.0 to 11.0 with 1 pH unit interval. The buffers used were 50 mM citrate buffer for pH range of 3–6, 50 mM sodium phosphate buffer for pH range of 6–8, and 50 mM glycine NaOH buffer for pH range of 8–11. XynA_MG1_ activities were determined using the standard enzyme assay, and a pH versus enzyme activity profile was plotted [19]. The enzyme stability at different pH was determined by preincubating XynA_MG1_ in different buffers from pH 3.0 to 11.0 (with 1 pH unit interval) for 30 min and 60 min at 25°C. Then, the remaining activities of the enzyme were measured under standard condition [20].

The optimum temperature for xylanase activity was determined by assaying the enzyme activity at different temperatures from 20 to 100°C with step increment of 5°C. Thermal stability was determined by incubating the enzyme in pH 5.5 buffer in the absence of substrate at the temperature range of 45 to 70°C. Then, the residual activities were measured under standard conditions [21].

### 2.6. Effect of Metal Ions, Salt, Chemical Agents, and Solvents on XynA_MG1_ Activity

XynA_MG1_ was incubated with 2 mM and 10 mM solution of Mn^2+^, Ca^2+^, Zn^2+^, Co^2+^, Cu^2+^, Mg^2+^, or nonmetal reagents including ethylenediaminetetraacetic acid (EDTA), dithiothreitol (DTT), *β*-mercaptoethanol (*β*ME), and detergents Triton X-100, Tween 80, and SDS, for 1 h at room temperature. Residual activity was measured under standard condition [22].

To investigate the impact of sodium chloride on XynA_MG1_ activity, the purified XynA_MG1_ was incubated with 0–4 M NaCl for 0 and 2 h and then assayed under standard conditions in the reaction containing NaCl at the same concentration as previously incubated [23].

XynA_MG1_ was incubated in aqueous solution of different solvents (acetone, methanol, ethanol, and propanol) at different concentrations (0–5 M) for 30 min. Residual activity was determined by using assay system under optimal conditions [22].

The nucleotide sequence of the* xynA*_*MG1*_ gene reported in this paper has been deposited in the GenBank database with an accession number of KX347434.

## 3. Results and Discussion

### 3.1. In Silico Sequence and Structural Analyses of XynA_MG1_ Xylanase

An* E. coli* clone harboring DNA from chicken cecum metagenome was found to contain an open reading frame of 1,116 bp encoding a protein of 372 amino acids, sequence of which was related to xylanase family GH10 and it was named the XynA_MG1_ xylanase. The first 23 N-terminus amino acids were predicted to be the signal peptide which guides secretion of the 349 amino acids' mature enzyme having a molecular mass and a pI value of 40,013 Da and 5.76, respectively.

**Figure 1:**
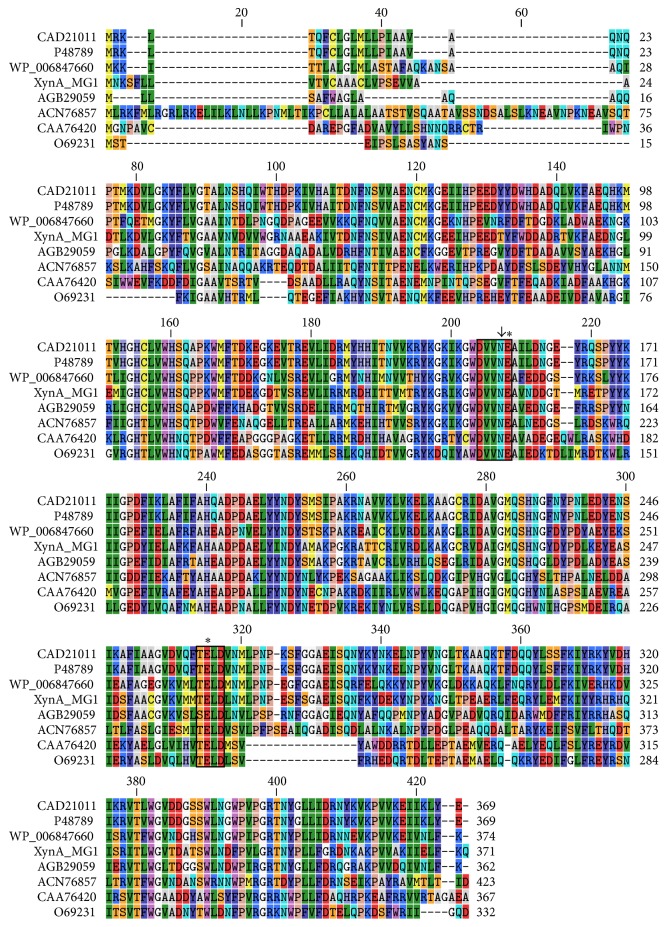
Amino acid sequence alignment of XynA_MG1_ with closely related GH10 family beta-xylanases from* Prevotella bryantii* B14 (CAD21011),* Prevotella ruminicola* B(1)4 (P48789),* Prevotella copri* DSM 18205 (WP_006847660),* Prevotella dentalis* DSM 3688 (AGB29059),* Paraglaciecola mesophila* KMM 241 (ACN76857),* Thermobacillus xylanilyticus* D3 (CAA76420), and* Paenibacillus barcinonensis* BP-23 (O69231). The highly conserved motifs DVVNE and TEXD are shown in boxes, each containing an active site glutamate (marked with an asterisk) and an invariant asparagine residue (marked with an arrow) preceding the active site glutamate in the first box.

Multiple sequence alignment of the XynA_MG1_ protein (including the signal peptide) with similar proteins, BLASTed and retrieved from the GenBank, revealed the highest identity at 59% to those of endo-1,4-beta-xylanases from* Prevotella bryantii* and* Prevotella ruminicola* and 58% identity to that from* Prevotella copri*. XynA_MG1_ has two conserved motifs, DVVNE and TEXD, of the GH10 family xylanase which contained the two glutamate residues (E157 and E 262) predicted to be the catalytic sites, and an invariant asparagine (N156) preceding the glutamate in the first motif serving as an acid/base catalyst (Figure 1) [24–26].

**Figure 2:**
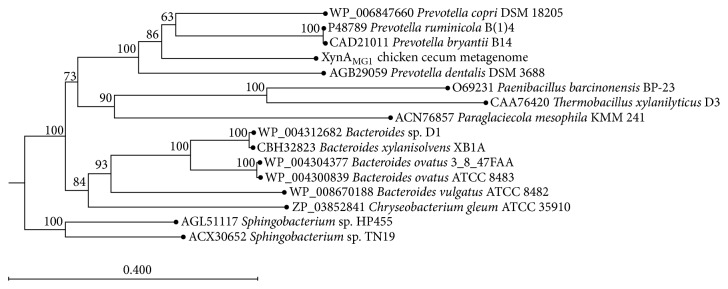
Phylogenetic analysis based on the amino acid sequence of XynA_MG1_ and related GH10 family xylanases. The phylogenetic tree was constructed using the neighbor joining method (CLC Main Workbench version 7.7). The lengths of the branches indicate the relative divergence among amino acid sequences. The percentage bootstrap values based on 1,000 bootstrap replications are shown at the nodes. Xylanases of* Sphingobacterium* sp. TN19 and* Sphingobacterium* sp. HP455 are used as outgroups. Accession numbers of the xylanase amino acid sequences are shown with the names of the host organisms. The scale bar represents the number of changes per amino acid position.

Phylogenetic analysis of XynA_MG1_ placed it in the cluster within* Prevotella *xylanases clade with high bootstrap value support (Figure 2). Among these, the highest amino acid sequence identity was only 59% which was the xylanase from* Prevotella bryantii* and* Prevotella ruminicola*. This indicated that XynA_MG1_ is a distinctly new endo-1,4 *β*-xylanase member of the family GH10, probably from a bacterium in the genus* Prevotella*.

**Figure 3:**
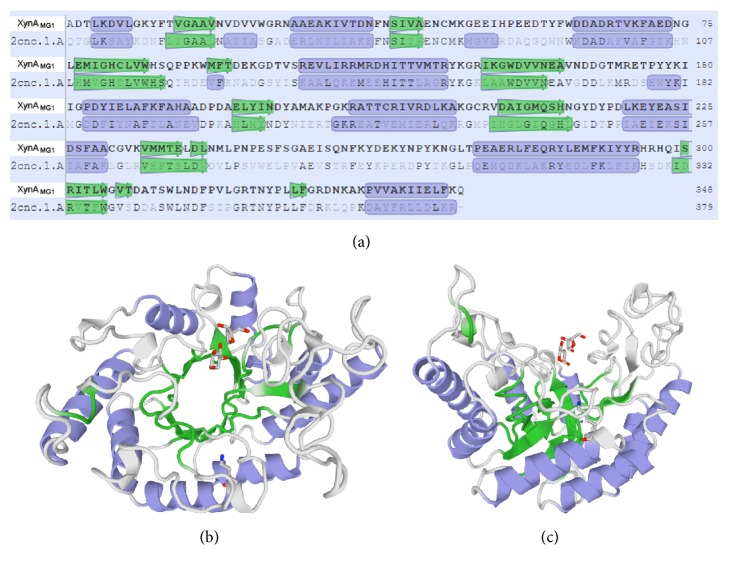
Structure of XynA_MG1_ modeled using crystal structure of a closely related GH10 endo-xylanase (2cnc.1.A) as a template. (a) Predicted secondary structure of XynA_MG1_; purple box: alpha-helix; green arrow: beta-sheet. (b, c) Three-dimensional representation of XynA_MG1_ with alpha-helices, beta-sheets, and loops folded into the typical (*β*/*α*)_8_ TIM-barrel structure; (b) top view; (c) side view. The XynA_MG1_ xylanase is shown with 2 molecules of *β*-D-xylopyranose (stick models) on the catalytic face.

Structure of XynA_MG1_ was modeled [27] using the protein crystal structure 2cnc.1.A of a closely related thermostable GH10 xylanase from* Cellvibrio mixtus* as a template. The amino acid sequence stretches in the XynA_MG1_ molecule predicted to form different secondary structures (alpha-helices, beta-sheet, and loops) are shown in Figure 3(a). Despite the low degrees of amino acid sequence homology of the mature forms of the two enzymes (40.2% identity; 58.6% similarity), the secondary structure profiles at corresponding positions along the amino acid sequences of the two xylanases are highly similar (Figure 3(a)). Three-dimensional structure modeling of XynA_MG1_ revealed the typical (*β*/*α*)_8_ TIM-barrel fold resembling the shape of a salad bowl (Figures 3(b) and 3(c)) which is similar to other known GH10 family xylanases [24].

### 3.2. Cloning, Expression, and Purification of XynA_MG1_ Xylanase

**Figure 4:**
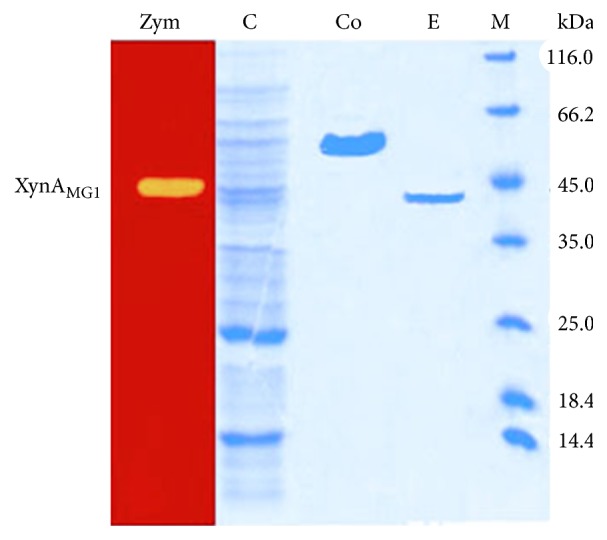
Purification steps of XynA_MG1_ and zymogram analysis. Proteins were separated with 12% gel SDS-PAGE. Lane (Zym) zymogram, lane (C): total soluble cell lysate from* E. coli* host expressing XynA_MG1_, lane (Co): Trx-His-XynA_MG1_ fusion protein purified by TALON cobalt resin, lane (E): purified XynA_MG1_ after digestion with enterokinase, and lane (M): protein molecular weight markers.

XynA_MG1_ without the signal peptide was expressed in* E. coli* with thioredoxin A peptide and hexa-histidine tags fused to its N-terminus to enhance soluble protein folding and facilitate protein purification, respectively. The purified fusion protein was digested with enterokinase to remove the peptide tags and subjected to second round of purification to yield the purified mature XynA_MG1_ enzyme for characterization experiments. The Trx-His-XynA_MG1_ fusion enzyme was catalytically active; however, its activity was around half of that of the mature XynA_MG1_ (data not shown).  Figure 4 shows the SDS-PAGE analysis of different forms of the enzyme through successive purification steps. The molecular mass of the purified XynA_MG1_ after removal of the tags was determined to be around 41.0 kDa which was close to the theoretical value of 40.013 kDa calculated based on the amino acid sequence. Xylanase zymogram analysis displayed a clear band of xylan digestion corresponding with the position of the XynA_MG1_ protein band (Figure 4).

### 3.3. Substrate Specificity

The purified XynA_MG1_ was found to exert its highest hydrolytic activity towards the beechwood xylan while the activity towards oat-spelt xylan was 94% relative to that of the beechwood xylan. No activity could be measured with *α*-cellulose, carboxymethyl cellulose, starch, and *β*-glucan as substrates. Upon testing with synthetic chromogenic substrates, XynA_MG1_ could hydrolyze only the 4-nitrophenyl-*β*-D-xylopyranoside while 4-nitrophenyl-*β*-D-cellobioside or 4-nitrophenyl-*α*-D-galactopyranoside could not serve as substrates (Table 1). The relatively specific xylanase property of XynA_MG1_ is in contrast with most xylanases of family 10 which have both xylanase and cellulase activities. However, a number of xylanases of family 10 showed only xylanase activity such as xylanase Xyn10N18 derived from a bovine rumen metagenomic library [28] and Xyn10J from a compost metagenomic library which showed negligible hydrolytic activity against carboxymethyl cellulose [18]. Added to the latter group is XynA_MG1_ isolated in this study and the possession of the cellulase-free xylanase activity makes it potentially very useful for biobleaching of pulps [18].

**Figure 5:**
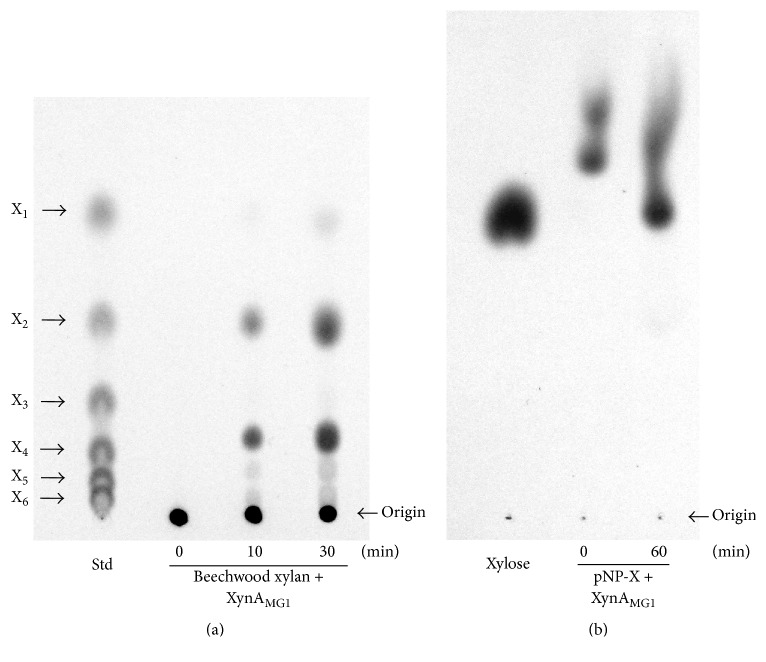
Thin-layer chromatography analysis of end products of XynA_MG1_ catalyzed hydrolysis of beechwood xylan (a) and 4-nitrophenyl-*β*-D-xylopyranoside (b). X_1_, xylose; X_2_, xylobiose; X_3_, xylotriose; X_4_, xylotetraose; X_5_, xylopentaose; X_6_, xylohexaose; Std, molecular weight standard mix of X_1_ to X_6_; pNP-X, 4-nitrophenyl-*β*-D-xylopyranoside.

TLC analysis of the reaction mixture of XynA_MG1_ catalyzed hydrolysis of beechwood xylan, compared with the results reported for the novel family 10 xylanase Xyn10J from a compost metagenomic library [18], suggested that xylobiose (X_2_) and xylotetraose (X_4_) were two major end products, and xylose (X_1_) was produced as a minor product (Figure 5(a)). XynA_MG1_ showed high hydrolytic activity on 4-nitrophenyl-*β*-D-xylopyranoside (pNP-X), a synthetic chromogenic substrate, releasing equimolar of two products 4-nitrophenol and xylose. The extent of the reaction is determined by adding 1 mL of 1 M Na_2_CO_3_ to stop the reaction and turn the 4-nitrophenol to yellow color which was quantified by measuring the absorbance at 400 nm [17]. The present xylose as a reaction product was shown by TLC (Figure 5(b)). XynA_MG1_ effectively digested the synthetic substrate 4-nitrophenyl-*β*-D-xylopyranoside releasing large amount of xylose. However, upon digestion of beechwood xylan, a natural substrate, the major end product was not xylose (X_1_) but the xylotetraose (X_4_) and xylobiose (X_2_) suggested that XynA_MG1_ could catalyze transglycosylation reactions. Xylanases with transglycosylation activity have been previously reported in the GH10 family, such as Xyn10J from compost metagenome and XynB from* Thermotoga maritima* and several GH11 xylanases such as Xyn1 from* Paenibacillus* sp. W-61, TfxA from* Thermomonospora fusca*, AnxA from* Aspergillus niger*, and XynA from* Paecilomyces thermophila *[18]. With the transglycosylation activity, XynA_MG1_ can be useful in the application for the synthesis of alkyl oligoglycosides [18].

### 3.4. Effects of pH and Temperature on Enzyme Activity and Stability

**Figure 6:**
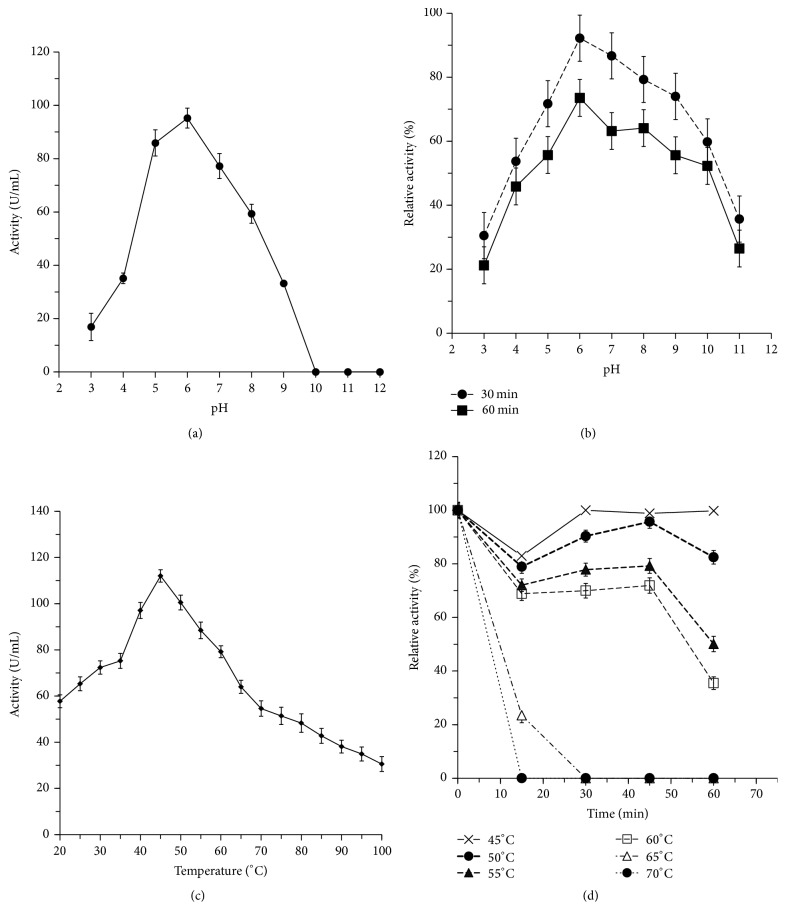
Effects of pH and temperature on the activity and the stability of the purified XynA_MG1_. (a) Effect of pH on activity at pH 3.0 to 11.0. The maximum activity was detected at pH 5.5 and was taken as 100%. (b) The pH stability of XynA_MG1_, incubated at pH 3.0 to pH 11.0 for 30 min and 60 min, at 50°C. (c) Effect of different temperatures on the activity of XynA_MG1_. The maximum activity was detected at 45°C. (d) Thermal stability after incubation at 45°C to 70°C for various times. The data were presented as mean ± SD (*n* = 3).

The purified XynA_MG1_ showed the typical bell-shaped pH profile with an optimal pH of 6. It retained 32% of its initial activity at pH 9 and completely lost all of its activity at pH 10 (Figure 6(a)). This is consistent with xylanases previously reported [26, 29] and with the physiological function of xylanases in the cecum of broiler chickens since the pH values in cecum broiler chicken are generally 5.5-6 [30]. The xylanase XynA_MG1_ enzyme was stable between pH 5.0 and 8.0 for 60 min, retaining around 70% of its activity at pH 8.0 (Figure 6(b)). It was fairly stable at high pH similar to other bacterial xylanases [26, 31].

The optimal temperature of XynA_MG1_ was 45°C (Figure 6(c)), which was close to those found in other metagenomic family 10 xylanases [26, 29]. Most xylanases of family 10 were known to have optimum temperature of 40–80°C [32]. XynA_MG1_ retained 72% of its activity after incubating at 60°C for 45 min (Figure 6(d)). However, when the temperature was 70°C, the enzyme completely lost the activity. Interestingly, it had around 30% activity even at 100°C in the presence of the substrate (Figure 6(c)). Many enzymes were known to be more stable in the presence of their substrates [33, 34].

### 3.5. Effect of Metal Ions and Chemicals on the Xyn_MG1_ Activity

As shown in Table 2, the XynA_MG1_ activity was slightly deactivated by metal ions Co^2+^, Zn^2+^, Mn^2+^, and Mg^2+^ at 2 mM and 10 mM in a concentration-dependent manner. Moderate inhibitions were found with Ca^2+^ and strong inhibitions were found with Cu^2+^. The inhibitions of xylanase activity by Cu^2+^ ion were commonly reported [35, 36].

Reducing agents such as *β*-mercaptoethanol and DTT slightly affected the XynA_MG1_ activity suggesting that xylanase does not seem to need disulfide bonds to achieve the hydrolysis reaction [37].

XynA_MG1_ enzyme retained more than 87% of its activity after incubation for 60 min with 10 mM of EDTA. The resistance of XynA_MG1_ to the chelating reagent suggested that it is not a metalloenzyme and no metal ion is essential for XynA_MG1_ activity. Its stability against the chelating agent, one of the indispensable ingredient in detergent formulations, is of great importance and worth further investigation on its application.

The anionic surfactant SDS which is well-known to cause protein denaturation severely deactivated XynA_MG1_. Many xylanases were known to be strongly affected by SDS. These include xylanase rMxyl from compost-soil metagenome, xylanase from* Burkholderia* sp. DMAX,* Aspergillus awamori *VTCC-F312, and* Aspergillus giganteus* [9, 38–40]. This indicated that hydrophobic interactions are important in maintaining the structure of XynA_MG1_.

Nonionic surfactants like Triton X-100 and Tween 80 at 2 mM showed mild inhibitory effects. However at 10 mM, they slightly enhanced the XynA_MG1_ activity which are in good agreement with other recombinant xylanases [41].

### 3.6. Effects of Sodium Chloride

**Figure 7:**
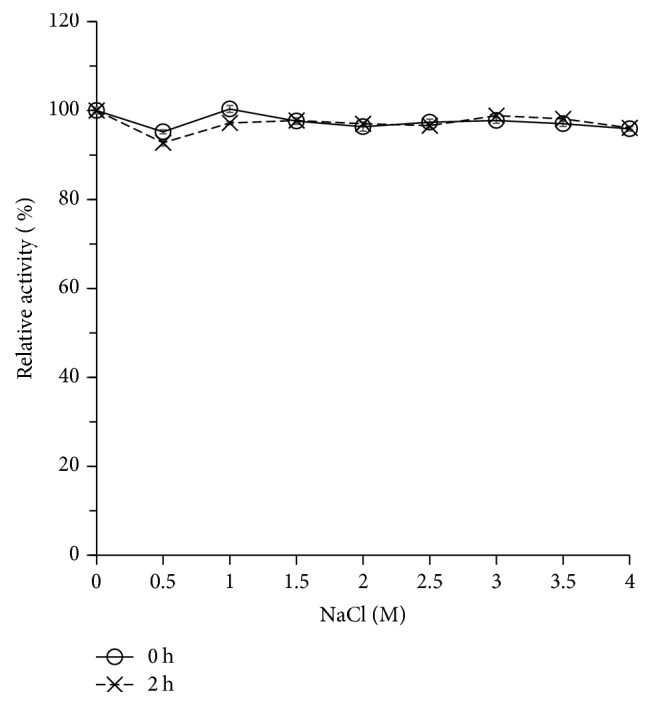
Effects of NaCl on XynA_MG1_ activity and stability. The xylanase activity at the starting time and without NaCl added was taken as 100%. The data were presented as mean ± SD (*n* = 3) (for experimental details, please see the main text).

Purified XynA_MG1_ showed excellent salt tolerance. While NaCl was not required for its xylanase activity, XynA_MG1_ could function at more than 96% of its activity in the presence of 1 to 4 M NaCl for at least 2 h (Figure 7).

High salt-tolerant and halophilic xylanases have been reported. Recently, a xylanase from* Massilia* sp. RBM26 isolated from feces of* Rhinopithecus bieti* was reported to maintain around 86% activity in 5 M NaCl for 1 h [26]. Xylanase from* Aspergillus giganteus* was shown to tolerate to up to 20% (3.4 M) of NaCl [38]. A cold-active xylanase from* Glaciecola mesophila* KMM 241 exhibited its highest activity at 0.5 M NaCl and retained 90% of the activity in 2.5 M NaCl at its optimal temperature of 30°C [35]. A xylanase from a marine bacterium* Bacillus subtilis *cho40 was reported to be greatly activated to 140% when preincubated with 0.5 M NaCl for 4 h [19]. A cold-active and halo-tolerant Xyn10A xylanase from* Bacillus* sp. SN5 was reported to exhibit the highest activity (134%) in 0.5 M NaCl and retain 90% activity in 2.5 M NaCl [42]. In comparison to the above-mentioned xylanases, our XynA_MG1_ reported here has the highest salt-tolerance property.

### 3.7. Effects of Organic Solvent on Xylanase XynA_MG1_ Activity

**Figure 8:**
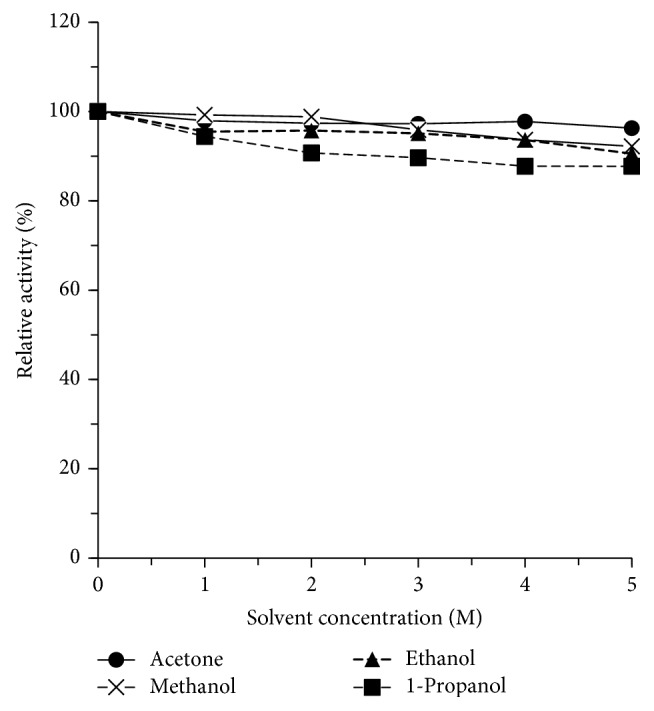
Effects of water-miscible organic solvent on XynA_MG1_ xylanase activity and stability. The xylanase activity at the starting time and without organic solvent added was taken as 100%. The data were presented as mean ± SD (*n* = 3) (for experimental details, please see the main text).

The XynA_MG1_ activity was only slightly affected by common water-miscible organic solvents like acetone, methanol, ethanol, and 1-propanol at the concentration of up to 5 M (Figure 8). At the highest concentration tested (5 M), acetone and methanol showed nearly no effect on XynA_MG1_ activity which is similar to the xylanase from* Streptomyces rameus* L2001 [43]. Comparing among alcohols, 1-propanol and ethanol which have lower polarity index values (and thus more hydrophobic) than methanol slightly inhibited XynA_MG1_ by 10% and 14%, respectively. These were similar to those reported for a xylanase (XynA) from* Clostridium cellulovorans* [44]. Hydrophobic interactions with the dissolved organic solvent molecules seem to be the major factor affecting the protein stability and the enzyme activity in this case [45].

In addition to microbial xylanases mentioned in the above discussions, biochemical properties of bacterial GH10 xylanases especially those from animal intestines are listed in Table 3 for comparison with the XynA_MG1_ xylanase described in this study. XynA_MG1_ is shown to be better tolerance to organic solvent and high salt (NaCl) concentration than other xylanases compared.

## 4. Conclusion

XynA_MG1_ derived from a chicken cecum metagenome is a new member of the GH10 family xylanase related to the xylanases from* Prevotella* sp. It is resistant to metal ions, reducing agents, and certain detergents, and highly tolerant to high concentrations of salt and water-miscible organic solvents. These make XynA_MG1_ a potential candidate for applications in the enzymatic processes operated at high concentration of salt and organic solvent such as in food and biofuels industries.

The salt-tolerant xylanase property of XynA_MG1_ can be useful in the processing of sea food and food with a high salt content which contain 0.5 to 2.5 M NaCl, such as marine algae, pickles, and sauce [35]. Furthermore, food materials washing, food processing, and fermentation under high salt condition could reduce cost because sterilization is not required [26]. In bakery industry, salt-tolerant XynA_MG1_ xylanase has potential applications in increasing the strength of the dough and adding flavor to baked goods [52].

Modern biofuel (bioethanol) production from lignocellulose biomass employs consolidated bioprocess where both the saccharification (enzymatic hydrolysis of cellulose and hemicellulose components to simple sugar) and fermentation steps take place within the same bioreactor, which makes the process more economical [44]. Tolerance to the carried over biomass pretreatment agents (acid, alkali, and inhibitors) and ethanol tolerance have been identified as two key elements for the enzymes in this consolidated process. The biomass saccharification enzymes, including xylanase, must be able to withstand and function well in the presence of 23 to 63 g/L (0.5–1.4 M) ethanol produced from the fermentation step. XynA_MG1_ can be a potential candidate for this application as it could tolerate up to 5 M ethanol.

XynA_MG1_ functions optimally at the temperature and the pH range of the chicken intestine, its native environment where its gene was retrieved by metagenomic cloning. This xylanase has potential utility in animal feed to improve nutrient digestibility and growth performance for animals, especially broiler chickens and hens [53].

## Figures and Tables

**Table 1:** Substrate specificity of the purified recombinant XynA_MG1_

Substrate^a^	Relative activity (%)^b^
Beechwood xylan	100
Oat-spelt xylan	94
Carboxymethyl cellulose	0
*α*-Cellulose	0
Starch	0
Barley *β*-glucan	0
4-nitrophenyl-*β*-D-xylopyranoside	55
4-nitrophenyl-*β*-D-cellobioside	0
4-nitrophenyl-*α*-D-galactopyranoside	0

^a^The test concentration for polysaccharide substrates was 1% (w/v) while that for the synthetic chromogenic substrates was 4 mg/mL. ^b^The activity towards beechwood xylan which was the highest activity was defined as 100%. All the values are means of three replications.

**Table 2:** Effect of metal ions, reducing agents, and ionic and nonionic surfactants on XynA_MG1_ xylanase activity.

Agent	2 mM	10 mM
None	100 ± 0.2	100 ± 0.2
Mg^2+^	96.8 ± 0.9	92.4 ± 0.7
Ca^2+^	91.4 ± 3.1	77.3 ± 2.9
Mn^2+^	99.9 ± 2.5	97.3 ± 3.7
Co^2+^	98.5 ± 2.9	94.2 ± 2.2
Cu^2+^	86.3 ± 2.2	41.4 ± 1.9
Zn^2+^	98.8 ± 0.9	95.5 ± 1.4
*β*-Mercaptoethanol	95.3 ± 1.0	93.6 ± 1.1
DTT	95.0 ± 1.6	94.7 ± 2.0
EDTA	91.6 ± 3.1	87.3 ± 3.1
SDS	22.1 ± 1.5	14.7 ± 1.3
Triton X-100	96.2 ± 2.2	103.9 ± 1.2
Tween- 80	97.9 ± 2.8	108.8 ± 2.6

The purified XynA_MG1_ was assayed in the standard assay condition in the presence of 2 mM or 10 mM test agents. The xylanase activity measured in the absence of the test agent was set as 100%. All the values are means of three replications.

**Table 3:** Biochemical properties of GH10 xylanases from animal intestinal bacteria compared with XynA_MG1_.

Source	Xylanase name	Optimal		Stability [residual activity (%)]		Tolerance [residual activity (%)]		Reference
		Temp (°C)	pH	Temp	pH	Solvent	NaCl salt	
*Microbacterium trichothecenolyticum *HY-17 from *Gryllotalpa orientalis* gut	rXylH	60	9.0	55°C, 30%	pH 5.5–10, 80%	ND	ND	[46]

*Paenibacillus macerans* IIPSP3 from termite gut	IIPSP3	60	4.5	90°C, 70%	pH 3.5, 40% pH 9.5, 67%	ND	10 mM, 119%	[47]

*Sphingobacterium* sp. TN19 from *Batocera horsfieldi* larvae gut	XynA19	45	6.5	40°C, 90%	ND	ND	10 mM, 95%	[48]

*Cellulosimicrobium* sp. HY-13 from earthworm gut	XylK	55	6.0	ND	ND	ND	ND	[49]

*Massilia* sp. RBM26 from *Rhinopithecus bieti* feces	XynRBM26	45	5.5	30–50°C, 62%	pH 5.5–10.0, 80%	ND	5 M, 86%	[26]

*Caldicellulosiruptor bescii *from geothermally heated freshwater pool	CbXyn10B	70	7.2	60–75°C, 60%	pH 8.0, 50%	ND	ND	[50]

*Bacteroides xylanisolvens* from human gut	XB1A	37	6.0	48°C, 80%	pH 9.0, 50% pH 5.0, 50%	ND	ND	[51]

Chicken gut metagenome	XynA_MG1_	45	6.0	60°C, 72%	pH 3, 25% pH 8, 70%	5 M acetone, 98% 5 M ethanol, 86%	4 M, 96%	This study

ND = not determined.
